# Gestational Viral Infections: Role of Host Immune System

**DOI:** 10.3390/microorganisms11071637

**Published:** 2023-06-22

**Authors:** Silvia Beltrami, Sabrina Rizzo, Giovanna Schiuma, Giorgia Speltri, Dario Di Luca, Roberta Rizzo, Daria Bortolotti

**Affiliations:** 1 Department of Chemical, Pharmaceutical and Agricultural Science, University of Ferrara, 44121 Ferrara, Italy; silvia.beltrami@unife.it (S.B.); sabrina.rizzo@unife.it (S.R.); giovanna.schiuma@unife.it (G.S.); giorgia.speltri@edu.unife.it (G.S.); brtdra@unife.it (D.B.); 2 Department of Medical Sciences, University of Ferrara, 44121 Ferrara, Italy; ddl@unife.it

**Keywords:** pregnancy, viruses, immune system, DNA viruses, RNA viruses

## Abstract

Viral infections in pregnancy are major causes of maternal and fetal morbidity and mortality. Infections can develop in the neonate transplacentally, perinatally, or postnatally (from breast milk or other sources) and lead to different clinical manifestations, depending on the viral agent and the gestational age at exposure. Viewing the peculiar tolerogenic status which characterizes pregnancy, viruses could exploit this peculiar immunological status to spread or affect the maternal immune system, adopting several evasion strategies. In fact, both DNA and RNA virus might have a deep impact on both innate and acquired immune systems. For this reason, investigating the interaction with these pathogens and the host’s immune system during pregnancy is crucial not only for the development of most effective therapies and diagnosis but mostly for prevention. In this review, we will analyze some of the most important DNA and RNA viruses related to gestational infections.

## 1. Introduction

Pregnancy is characterized by a distinct immunological status that is designed to protect the fetus from maternal rejection but also to allow an appropriate fetal development and defense against pathogens [1,2].

The placenta represents the interface through maternal and fetal tissues, in which immunological changes occur that permit rejection avoidance of the semi-allogenic fetus by the maternal immunity and, at the same time, protect the fetus from viral infections until its birth [3].

The placental innate immune response to pathogens is important to protect both the fetus and mother. Thus, impairments of these immune effectors could allow the development of infections that might be associated to several diseases in pregnancy. The potential capacity of pathogen patterns’ recognition and other host defense strategies have been described in the innate immune cells at the placental–decidual interface [4].

The protective effect of placenta toward infection is also due to its ability to transmit maternal protective antibodies to the fetus through the chorionic villus of the syncytiotrophoblast (SCT). Maternal Immunoglobulin G (IgG) passage to fetal circulation is mediated by neonatal FcRn receptors (FcgammaRI, FcgammaRII and FcgammaRIII) expressed on placental macrophages [5], and their concentrations in fetal blood increase from the beginning of the second trimester until the end of pregnancy.

The peculiar immunological status observed during pregnancy is characterized by an increase in circulating immune cells, which reaches the peak in the second trimester. During the first trimester, Foxp3+ regulatory T (T-reg) cells, essential for healthy gestational development and establishment of the tolerogenic status, are inducted by estrogen [6,7,8] and found to be abundant in peripheral, deciduous and umbilical cord blood [9]. Here, T-reg participates in the control of CD4+ and CD8+ T lymphocyte activation via Interleukin-10 (IL-10) and Transforming Growth Factor (TGF) release [10]. In fact, hormonal changes represent an important factor which affects immune responses during pregnancy, which might result in a decrease in the number of dendritic cells (DCs) and monocytes as well as a decrease in the activation of macrophages, T cells and B cells [11]. In fact, compared to the postpartum period, CD4+ and CD8+ T cells activation is down-regulated and, particularly during the third trimester, B lymphocytes are reduced [12].

Another notable aspect concerns the natural killer (NK) cells, lymphocytes of the innate immune system that function as the first line of defense against viral infection, which are found increased in maternal blood during the first trimester [13] and can account for up to 70% of the total deciduous leukocytes in early pregnancy [14]. In this contest, NK cells are specifically named decidual NK cells (dNK); they have a significant role in the regulation of cytokine production, particularly IL-10, as well as the generation of angiogenic factors and chemokines, which is crucial to control trophoblast invasion and vascularization at the implantation site [14,15,16]. Importantly, during the second and third trimesters of pregnancy, cytotoxic CD56 dim NK cells are lower than in the first trimester and postpartum period. In particular, NK cells shift their phenotype toward a more secretory profile, depicted as an increase in CD56 expression (NK CD56 bright), and produce less Interferons (IFNs), Tumor Necrosis Factors (TNFs) and IL-6 [17,18] compared to the postpartum period. 

Of course, the activation of dNK toward viral infections reduces the potential risk of vertical transmission to the fetus. dNKs have been proposed to play a protective role against several infection through several mechanisms including the modulation of their cytotoxic effector functions [19] and the interactions between the killer-cell immunoglobulin receptors (KIRs) expressed by dNK and HLA molecules on the surface of the infected cells [20,21].

Hence, this implies that in the decidua, dNKs can eliminate harmful infection depending on the combination of KIR/HLA interactions between dNK and infected cells.

Again, concerning maternal monocytes, despite there being no evident differences in their total number, they exhibit phenotypic modifications, such as an increased expression of adhesion molecules (CD11a, CD54) and a high-affinity to IgG receptor FcR-I (CD64) [22]. In particular, Hofbauer (HB) cells, the fetal macrophages of the human placenta [23], can be detected as early as 3 weeks post-conception and are present throughout pregnancy [24]. Despite their role in villous and trophoblast remodeling [25], it has been postulated that HB cells may have a role in controlling infection during pregnancy, even if whether the HB cells can serve as a reservoir or limit virus replication is still unknown. As a proof of concept, isolated HB cells from healthy term placenta secreted elevated pro-inflammatory cytokines such as IL-6, MCP-1, IP-10, and IFN-α upon in vitro infection with Zika Virus (ZIKV) [26], even if HB cells are permissive for ZIKV infection and replication [27,28,29]. 

Blood-borne viruses can potentially be transmitted through the SCT barrier, even if SCT expresses only a few viral entry receptors. For example, SCT does not express ZIKV entry receptors (Axl and Tyro3) [30] and the Cytomegalovirus (CMV) entry co-receptor integrin α/β [31]. On the other hand, the STC apical surface expresses neonatal Fc receptor (FcRn) that functions to selectively transport maternal IgG [32], and it could be exploited by certain viruses to enter the placenta including ZIKV, Human Immunodeficiency Virus-1 (HIV-1), and CMV [33,34]. This FcRN-mediated viral entry at the STC, together with local inflammation and tissue damage, might disrupt its effective role as a barrier to most pathogens, giving the opportunity for vertical transmission.

However, humoral maternal response also actively participates, reducing the risk of vertical transmission, as supported by the low-affinity maternal antibodies that correlate with higher viral loads in the decidua, whereas intermediate to high neutralizing antibodies are associated with low viral replication [35].

Finally, also, a role in the development of placenta immune competence against the viral infection of both intra and extracellular-specific antiviral microRNAs has been suggested [36].

Clinical studies have shown that a high percentage of pregnancy complications, such as miscarriage, preterm delivery, growth retardation and preeclampsia, can be caused by intrauterine infections due to viruses, bacteria and other etiological agents [37]. The transmission of pathogens from mother to fetus can occur through several pathways: (a) via the maternal vascular endothelium to extravascular or endovascular trophoblasts (EVTs); (b) via infected macrophages in maternal blood that transmit the infection to placental trophoblasts; (c) via paracellular pathways from maternal blood to fetal capillaries [38,39]; and (d) via vertical transmission or ascending infection on urogenital tract [40]. The possible clinical consequences of maternal infection are explained in Table 1 [41].

## 2. Methods

From this perspective, we have reviewed the main current data referring to pregnancy in correlation with the immune system and infections as well as to signaling functions and the potential impact on clinical conditions. Data were selected following eligibility criteria according to the reviewed topic. We used a set of electronic databases (Medline/PubMed, Scopus, Web of Sciences (WOS), Cochrane Library) for a systematic search until May 2023 using MeSH keywords/terms, such as “pregnancy”, “viruses”, “immune system”, “DNA viruses”, and “RNA viruses”. We applied no date or language restrictions. We followed the Preferred Reporting Items for the Systematic Review and Meta-Analysis (PRISMA) statement [42]. Two independent reviewers performed title–abstract screening on all selected studies; then, the full texts of the selected articles were reviewed. In cases of duplicate information, the data were checked and combined. Studies reporting pregnancy as well as viral infections were selected. Publications were selected using specific keywords (i.e., pregnancy, infection, virus, etc.) also according to the date of publication (not older than 1988) and to guarantee the fulfillment of the topic of this review. Studies that were just case reports and commentaries were excluded. The extraction of the data from included studies was performed by two reviewers separately, considering key characteristics including publication year, author, type of study, country, sample size, and laboratory findings. The funnel plot and Egger’s regression test were used to assess publication bias [43].

Therefore, it is evident that infections could have several repercussions on both the embryo and the fetus. More specifically, in the first weeks of gestation, often before the woman becomes aware of the pregnancy, they can cause the death of the embryo, while after the first 6 to 8 weeks of gestation, the presence of a pathogen can interfere with fetal organogenesis or, in general, cause miscarriage or stillbirth. In many cases, early infections during pregnancy are associated with placental abnormalities, causing Intrauterine Growth Restriction (IUGR) [44]. On the contrary, infections in the third trimester are often responsible for premature births [45].

In addition, infections in newborns can be the cause of congenital diseases, due to the ability of microorganisms to survive and replicate in the tissues of infected infants, for months or years after infection in utero. In general, the presence of infectious agents and the release of endo- and exotoxins during pregnancy activate an immune response in both mother and fetus, triggering an inflammatory status due to the production of pro-inflammatory cytokines (TNFα, IL1-α, IL1-β, IL-6 and IL-8), which in turn stimulates chemotaxis, infiltration and the activation of other immune system cells [37,46].

Several studies have investigated the special immunological condition reported during physiological pregnancy, evidencing the importance of specific immune-regulatory processes, in which a crucial role belongs to the major histocompatibility complex molecules, in humans HLAs (Human Leukocyte Antigens), which are also involved in the response against viral infections [47,48]. The loss or down-regulation of classical HLA class-Ia antigens, as well as the neo-expression of non-classical HLA class-Ib antigens (HLA-E, -F, and -G), is a frequent strategy used by viruses for evading immune surveillance [49,50,51]. Among these non-classical HLA-Ib molecules, HLA-G and HLA-E represent the most studied for their tolerogenic role toward the fetus during pregnancy [52] and for their regulating function in neoangiogenesis during placentation [53]. 

HLA-G is an immunosuppressive molecule working through the interaction with specific inhibitory receptors (ILT2/LILRB1, ILT4/LILRB2 and KIR2DL4) expressed on immune cells, which leads to the inhibition of NK cells, CD8+ cytotoxic T lymphocytes and of macrophage-mediated cytotoxicity, allo-response, and maturation by CD4+ T cells, as well as the DCs functions. HLA-E is another crucial component of the immunological network at the fetal–maternal interaction [54]. HLA-E antigens have been discovered as ligands for a subset of immunoglobulin superfamily NK cell receptors CD94/NKG2, and their interaction may be responsible for the suppression of NK cell killer functions. In particular, peptide-loaded HLA-E binding to the inhibitory NKG2A receptor reduces NK cytokine cytotoxicity and secretion [55].

Again, the maternal HLA-C genotype seems to be crucial in defining uterine NK (uNK) cells function and maturation through interactions between maternal-activating or inhibitory killer immunoglobulin-like receptors (KIRs) expressed on the NK cell surface [56,57], and their peculiar expression has been reported to be associated to pre-eclampsia [58].

Viewing the role of HLA molecules in the antigen presentation process, several viruses are known to exploit classical and non-classical HLA-I regulation to avoid immune system recognition [59], including HHV-6 [60], HIV [61], CMV [62], Hepatitis B Virus (HBV) [63], Hepatitis C Virus (HCV) [64] and Human Papilloma Virus (HPV) [65].

Another important strategy adopted by viruses to evade their recognition by the host immune system consists of the escape from pattern recognition receptors (PRRs), which identified pathogen-associated molecular patterns (PAMPs), inducing inflammatory pathways by effectors of innate immunity [66]. Among the PRRs, Toll-like receptors (TLRs) are typically found on endosomal membranes [67], but this class of receptors is also expressed in the cellular cytoplasm or nucleus, which belongs to retinoic acid-inducible gene-I (RIG I)-like receptors (RLRs) [68]. Despite the activation of the PRRs pathway being enhanced and fundamental in pregnancy for viral infection clearance, the over-induction of the immune response initiated by PRRs might damage the surrounding tissues [69,70]. In fact, PRRs and mainly TLRs are expressed on trophoblasts, and they are also involved in placental damages, such as pre-eclampsia [71], preterm birth [72] and miscarriage [73]. Consequently, the lack of PRRs-mediated response might contribute to the fetal transmission of infection during pregnancy [74]. 

Therefore, both PRRs and HLA molecules may play protective and detrimental roles at the same time, potentially resulting in disorders linked to acute and chronic inflammation, since both DNA and RNA viruses often exploited the physiological tolerogenic condition that characterizes pregnancy as an immunological evasion strategy [75], facilitating the onset of complications in the pregnant woman and fetus.

## 3. DNA Viruses and Gestational Infection

Due to the immune-tolerogenic state characteristic of pregnancy, there are several infections that can occur in pregnant women, showing poor outcome. Among these, some of the most widespread infections are represented by DNA viruses. Similarly, to bacteria or RNA viruses, DNA viruses infections during pregnancy are associated with increased health risks to both mother and fetus. However, although not all DNA viruses are related to an increased risk of complications during pregnancy, several of them can directly infect the fetus and/or cause placental dysfunction. For this reason, it is important to be aware of the potential impact of these viruses on both mother and fetus health, on the maternal immune system and in their effect in pregnancy outcome.

### 3.1. Human Parvoviruses

Among gestational infections, it is possible to mention Human Parvoviruses, consisting of Parvo B19 Virus (B19V) and less frequently observed Adeno-associated viruses (AAVs) and Human Bocaviruses (HBoVs).

**Figure 1:**
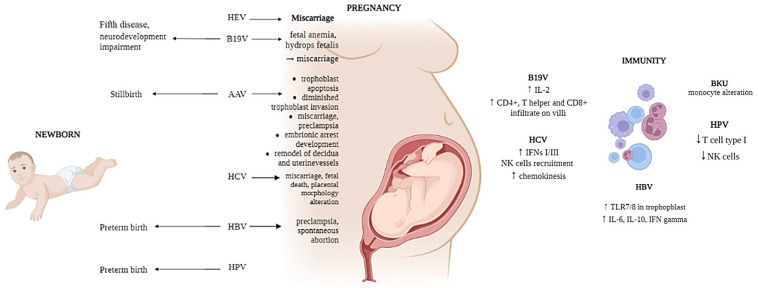
Representation of the main DNA viruses and their infection in newborn, pregnancy and mother immunity.

Although in healthy adults most B19V infections result in mild non-specific illness [76], B19V causes the fifth disease during childhood and persistent anemia in immunocompromised patients, such as pregnant women. During pregnancy, in fact, congenital B19V infection can be responsible for fetal anemia, leading to hydrops fetalis, increasing the risk of miscarriage (Figure 1). Nevertheless, even if fetal abnormalities as a result of congenital Parvovirus B19V infection are mostly uncommon [77], this infection could increase the risk of neurodevelopmental impairment [78,79] (Figure 1). Since it has been observed that B19V is capable of inducing hydrops fetalis, many studies focused on analyzing this phenomenon. Garcia et al. [80] described six cases of non-immune hydrops that were fetalis B19V-associated, pointing out the presence of a mononuclear cell infiltrate in the villous stroma and intervillous space in the whole cohort [80]. Nevertheless, it has been suggested that B19V infection is not only associated with an active maternal humoral immune response but also with an inflammation-mediated immune response at the maternal–fetal interface of the placenta [81]. However, although the presence of a lymphocyte infiltrate within placentas from women who became infected during pregnancy has been demonstrated, its specificity for B19V has not been evaluated yet [81]. 

Even if the cell-mediated immune response to placental Parvovirus infection is not well characterized, a high increase in IL-2 production has been highlighted in placentas from women with parvovirus B19 infections compared to placentas from healthy controls [81], and nearly 90% of the Parvovirus-infected placentas had either a CD4+ T-helper or CD8+ cytolytic infiltrate into the villi [81].

Among human Parvoviruses, it is possible to distinguish AAV as well. Among these, AAV2 and AAV3 are responsible for infecting humans, even if Adeno-associated viruses usually require co-infection with a helper virus (Adenovirus) to cause a productive infection. AAV2 can infect placental trophoblast cells, inducing placental dysfunction when the infection occurs in early pregnancy [82]. AAV2 also induces trophoblast apoptosis and reduces cell invasion [83,84], increasing the risk of spontaneous miscarriage, stillbirth and pre-eclampsia [84,85,86] (Figure 1). Moreover, in vitro studies demonstrated that AAV2 infection inhibits the development of mouse embryos [87], and in vivo studies of pregnant mice showed fetal demise after AAV2 infection [84]. All these results suggest the possibility that early embryonic and trophoblastic viral infection could hinder the implantation or placentation process, possibly by inducing an anti-trophoblast cellular immune response [85] (Figure 1).

### 3.2. Human Hepatitis Viruses

Additional DNA viruses often underestimated during pregnancy are Hepatitis B virus (HBV), Hepatitis C virus (HCV) and Hepatitis E virus (HEV).

HBV is a member of the Hepadnaviridae family that generally infects hepatocytes and causes inflammation of the liver [76] that could progress to chronic infection. Vertical transmission during birth leads to a high percentage of chronicity compared to a later infection, but the chances of mother-to-child transmission can be reduced by antiviral treatment during and after pregnancy [88]. The consequences of HBV infection during pregnancy are still unclear; however, research has shown that maternal HBV infection increases the risk of preterm birth [89]. HBV is one of the many viruses that have also been studied in the context of pattern recognition receptors (PRRs) signaling in trophoblasts. In particular, TLR7 and TLR8 expression in the placenta of HBV non-transmitted neonates was higher than in transmitted neonates [90]. In fact, HBV infection increased TLR7, TLR8, MyD88, IL8, IFN-α and IFN-β mRNA levels in Swan71 cells, supporting the capability of HBV to translocate across the placenta [90]. Therefore, the increase in TLR7/8 observed in trophoblast cells exposed to HBV suggests a role for TLR7/8 signaling in the prevention of maternal–fetal transmission (Figure 1). Moreover, HBV is also able to up-regulate IL-6, IL-10, and IFN-γ through its HBV-X protein in trophoblast cell line [91], consequently stimulating immune responses (Figure 1).

HCV is also a leading cause of hepatitis in the western world, and its vertical transmission shows a rate between 3% and 6% [92]. Since nowadays, there are currently no vaccinations for HCV and no preventive strategies to reduce the risk of vertical transmission, its gestational infection should not be underestimated.

In a cohort made up of 145 HCV-positive pregnant women, 3.4% experienced intrauterine fetal death, which was higher than the miscarriage rate in the general population (0.5%) [93], although little evidence supports the association between HCV and miscarriage (Figure 1). Since HCV receptors are expressed on the surface of trophoblasts and extravillous trophoblast (EVT) cells, these cells are susceptible to HCV infection leading to some gestational complications and have some impacts on the morphology of the placenta as well [94] (Figure 1). HCV vertical transmission seems also to be facilitated by the specific NK cell receptor repertoire. Khakoo et al. [95] found a correlation between KIR2DL3 receptor, an inhibitory receptor expressed on NK cells, and HLA-C1 on decidua in HCV infection resolution, but it is yet unclear if the mother’s HLA-C status at the maternal–fetal interface influences the decidual NK (dNK)-cell repertoire in HCV.

Some additional immune pathways that are stimulated by HCV infection and affect NK cells recruitment involve the up-regulation in Type I/III IFNs and other chemokines in trophoblast cells by HCV-RNA [74] (Figure 1). This causes the enhancement of dNK T cell and γδ T-cell cytotoxicity, resulting in damaging of the placental barrier (Figure 1). On the contrary, the reduced expression of NK cell activation markers CD69, NKp44 and TRAIL seems to improve HCV vertical transmission [96]. Nowadays, there are currently no vaccinations for HCV and no preventive strategies to reduce the risk of vertical transmission.

Concerning the Hepatitis E virus (HEV), its involvement in pregnancy could be attributed to hormonal and immunological changes that could make pregnant women more susceptible to HEV infection as well as a high viral load [97]. Pregnancy increases the levels of hormones including progesterone, estrogen and human chorionic gonadotropin, affecting the immune system function and in fact, estrogen and progesterone are reported to affect B-cell proliferation, CD8+ T-cell cytotoxicity and NK cells activation, promoting the spread of the infection [97,98]. The mechanisms underlying adverse fetal outcomes associated to HEV infection are largely unknown. However, an in vivo study conducted in HEV-infected pregnant mice showed a shift in the immune response from Th2 tolerogenic response, which is essential for maintaining pregnancy, to the pro-inflammatory Th1profile [97]. This immune response alteration has also been seen in HEV-infected pregnant women who have hepatic failure, which could be considered a biomarker for miscarriage [99] (Figure 1).

### 3.3. Human Papillomaviruses

Human papillomavirus (HPV) represents another group of important gestational infections. HPV mostly spreads by direct skin contact, but some cases of vertical transmission during pregnancy have also been reported [100]. Trophoblast cells have been shown to be susceptible to several HPV strains, which can consequently alter physiological placental function [101], inducing gestational complications such as preterm birth, pre-eclampsia and spontaneous abortion [102,103] (Figure 1). A higher incidence of cervical neoplasia has been found in immunosuppressed women [104] and therefore, this phenomenon could be observed in pregnancy as well, since it is a state of mild immunosuppression due to the reduction in the Th1 cell-mediated response or decrease in NK cells (Figure 1). Thus, pregnant women are characterized by a physiological state with an enhanced risk of HPV infection, which is followed by the development of neoplasia. Furthermore, since a steroidal hormone receptor binding element on the transcriptional promoter of HPV-16 is responsible for inducing HPV transcription, the involvement of hormonal activation of HPV replication has been suggested [105]. This condition supports that the altered immunity state and the increased steroidal hormonal levels during pregnancy might influence the subsequent progression of the disease development [105].

### 3.4. Human Polyomaviruses

Among the fourteen different species of Polyomaviruses known to infect humans, the most studied in association to gestational infection are BK virus (BKV) and JC virus (JCV) because of their capability to be vertically transmitted, representing a risk for both mother and fetus [106].

In particular, the detection of BKV is frequent in pregnant women with prevalence between 15% and 65% [107,108,109]. Recently, an increased BKV reactivation was shown during pregnancy [110,111], which might be due to multiple reasons, including immunological and hormonal changes happening in pregnant women. The development of tolerance toward the fetus, characterized by the physiological depression of cell-mediated immunity, is the main cause of reactivation of latent viruses during pregnancy, such as BKV. Moreover, changes in monocyte function during pregnancy seem to modulate the level of BKV reactivation as well [112] (Figure 1).

## 4. Human Herpesviruses

Human herpesviruses (HHVs) are highly widespread among humans and therefore are among the pathogens most responsible for gestational infections. HHVs are classified into three subfamilies (alpha-, beta- and gammaherpesvirinae), and they are able to establish permanent latency within the host in specific cells [113]. The alphaherpesvirinae family includes herpes simplex type-1 (HSV-1 or HHV-1), herpes simplex type-2 (HSV-2 or HHV-2) and varicella zoster virus (VZV or HHV-3). The betaherpesvirinae family includes cytomegalovirus (CMV or HHV-5), HHV-6A/B and HHV-7. The gammaherpesvirinae family consists of Epstein–Barr virus (EBV or HHV-4) and Kaposi’s sarcoma-associated herpesvirus (KSHV or HHV-8) [76].

### 4.1. HSV-1 and HSV-2

Normally, HSV-1 predominates in orofacial lesions and typically is found in the trigeminal ganglia, while HSV-2 is most found in the lumbosacral ganglia. The greatest risk of disease in the newborn is represented by the late-pregnancy infection of genitals in a previously unexposed woman, while recurrent infections are rarely associated with disseminated neonatal disease in immune-competent women.

However, a primary HSV infection of a pregnant woman leads to greater risks for both mother and child. Although HSV-infected pregnant women have rare or no clinical recurrences, there is still the risk of intrapartum transmission [114]. 

Women who already has antibodies to both HSV-1 and HSV-2 at the onset of pregnancy, which is the most common condition, have the least risk of perinatal transmission [115]. On the contrary, new-onset HSVs infection occurring late in pregnancy carries a 30% to 50% risk of neonatal infection, while early pregnancy infection carries a risk of less than 1% [116]. The possible explanation could be that when primary HSVs infection occurs during late pregnancy, the time for developing specific antibodies and suppressing viral replication before labor is not enough. 

**Figure 2:**
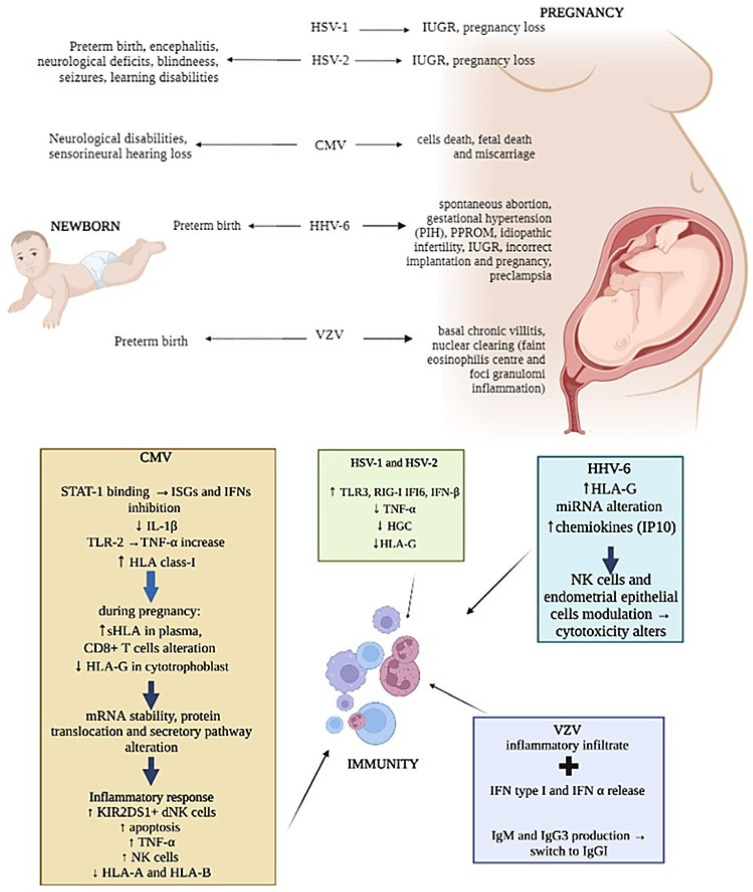
Representation of herpesviruses infection’s main effects on newborn, pregnancy and mother immunity.

Again, while HSV-1 transmission from mother to newborn seems to be easier in the presence of both primary infection or recurrences [117], primary HSV-2 infection transmission to the fetus is less frequent [118], but it has been associated with a higher incidence of preterm birth [119] (Figure 2). The clinical manifestations of neonatal HSVs infection include encephalitis and disseminated disease, with a mortality rate of more than 50%. Survivors are, however, compromised, usually with significant neurologic deficits, blindness, seizures and learning disabilities (Figure 2). Different studies have also stated that HSVs infection can affect maternal immune responses, resulting in loss of HLA-G [120], cell death and reduced human chorionic gonadotropin (HCG) secretion [121] (Figure 2). These changes in trophoblast function could explain why both HSV-1 and HSV-2 have been associated with spontaneous pregnancy loss [122] and IUGR pregnancies [85,123] (Figure 2).

Moreover, HSV is capable of increasing the expression of TLR3, RIG-I, IFI6 and IFN-β proteins as well as decreasing TNFα production in terms of human placental explant cultures [124] (Figure 2), affecting maternal innate immune system antiviral responses.

### 4.2. CMV

Among herpesviruses, CMV is one of the most vertically transmitted and it represents the most common cause of congenital infection in high-income countries, causing neurological disability and sensorineural hearing loss in newborns [125] (Figure 2). The intrauterine infection caused by CMV occurs in 0.3% to 2.3% of births [126]. CMV intrauterine transmission is more common after primary infection (30–40%) than after non-primary infection (1%) [127,128]. Nevertheless, it was estimated that non-primary maternal infections are responsible for the majority of congenital CMV infections [129]. 

CMV, such as many other viruses, employs multiple mechanisms to exploit the vulnerability of the placenta and impair the innate host response in order to spread the infection, including the expression of several viral proteins such as IE1 [130], IE86 [131], UL44, IE2 and UL94 [130,132,133]. 

The effect of CMV on the placenta has been confirmed by proving that ultraviolet-inactivated human CMV leads to syncytiotrophoblast apoptosis via TLR2, also increasing TNFα production [134,135]. Interestingly, in human villous explants, CMV did not induce the expression of RIG-I and MDA5 proteins and cytokine production [136], suggesting that RLRs could play a central role in the inhibition of vertical transmission of the virus as well as in the selectivity of vertical transmission of viruses across the placenta.

CMV can modulate the expression of HLA molecules by encoding specific viral proteins, mainly decreasing their expression on the cell surface to prevent immune cells recognition. In addition, CMV can selectively up-regulate specific HLA class-I molecules [137] (Figure 2). As aforementioned, HLA-G is physiologically expressed at the maternal–fetal interface on trophoblasts and is one of the major molecules targeted by viruses during pregnancy [138]. The concentrations of sHLA-G normally increases in the plasma of pregnant women during the first trimester of pregnancy [139], but during CMV infection, a reduction in HLA-G in cytotrophoblasts was observed [140] together with its up-regulation in peripheral blood cells [141] (Figure 2). This effect is due to the interaction of specific CMV proteins with the HLA-G promoter, which affects mRNA stability and protein translation and secretion [142,143,144] (Figure 2). 

Several studies have reported the failure of dNK to control CMV infection. Since NK cells activity is regulated by the expression of activation/inhibitory killer imuunoglubulin-like receptors (KIRs), such as KIR2DL4, KIR2DS1/5, etc. as activating, and KIR2DL1/2/3, etc. as inhibiting, the expression of these receptors and of their ligands on target cells can be exploited by viruses as immune-escape mechanisms. Crespo et al. [145] demonstrate that CMV infection of human HLA-C2 + decidual stromal cells drives the cytotoxic activation of dNK and placental NK (pNK) cells in vitro by engaging KIR2DS2, and that KIR2DS1 or KIR2DS5-negative pregnant women have a lower ability to control placental CMV infection, developing complications. Van der Ploeg et al. [21] reported the molecular basis for the increased degranulation response of KIR2DS1 + dNK to CMV infection (Figure 2). Yan et al. [146] show that a KIR2DL4/HLA-G combination induces high NK cytotoxicity, which might be beneficial uterine CMV infection. Other studies described the presence of adaptive NK cell expansion found during different viral infections, concluding that in CMV-infected individuals, adaptive NK cells may be established probably as the result of opportunistic viral reactivation [147].

Moreover, the interaction between CMV and dNK cells can be the cause fetal death or miscarriage due to NK cell cytotoxic activity [148] (Figure 2). However, the CMV infection of EVT did not diminish the ability of EVT to increase FOXP3+ and PD1HI T-regs [149], suggesting that its infection does not alter the capacity of EVT to promote immune tolerance. This finding confirms the observation that dNK fails to degranulate in response to CMV-infected EVT, thus also maintaining immune tolerance in the presence of infection [20].

Moreover, the failure of dNK to respond to CMV-infected EVT during in vitro co-culture [20] may leave decidual CD8+ T cells as the predominant effector cell to clear pathogen-infected EVT. 

Seropositive women during late pregnancy demonstrated an accumulation of highly differentiated CMV-specific T cells [150]. In fact, CMV seropositivity was shown to dramatically alter the maternal CD8+ T-cell repertoire during pregnancy [150], and T-cell responses to CMV rely heavily on HLA-C-restricted signals [151] (Figure 2). CMV CD8+ T cells were also found increased particularly in decidual tissue and were found able to produce IFNγ and restricted to recognizing viral peptides presented by HLA-A or HLA-B molecules, limiting the spread of infection to trophoblasts and/or the fetus [152].

### 4.3. HHV-6

HHV-6 is widely spread during pregnancy as well. HHV-6 DNA has been detected in blood and tissue samples from women with several types of gestational problems, including spontaneous abortions, gestational hypertension and preterm birth, in association with the detection of high anti-HHV-6 IgM and IgG titers [153] (Figure 2). HHV-6 DNA was also found in the amniotic fluid of women with gestational complications [154], as pregnancy induced hypertension (PIH) and the premature preterm rupture of membranes (PPROM) (Figure 2). 

To date, despite different congenital herpetic infections having been associated with late IUGR, no direct implication of HHV-6 infection has been reported. In particular, HLA-G expression and HHV-6 infection have been evaluated in placentas from late-onset IUGR newborns compared to placentas from uncomplicated pregnancies [155], since HHV-6 is known to exploit the modulation of HLA-G as an immune-escape mechanism. 

HLA-G increased and HHV-6 presence were found to correlate in IUGR placenta samples [155]. These preliminary results underline a direct relationship between HHV-6 infection and HLA-G deregulation that might affect vessel remodeling and prevent the correct pregnancy outcome in the IUGR condition (Figure 2).

However, HHV6 is comprised of two species, HHV-6A and HHV-6B [156,157]. While most of the population is infected by HHV-6B by 2 years of age, HHV-6A infection usually occurs later [158,159,160]. In particular, HHV-6A clinical manifestations are still unclear, but the presence of HHV-6A in endometrial epithelial cells of a subgroup of idiopathic infertile women [161] supported the role of HHV-6A [162] (Figure 2). 

Moreover, it has been observed that HHV-6A infection induces a profound remodulation of miRNA expression in human cells of different origin [163], including human endometrial cells, in which HHV-6A modulates at least 16 miRNAs with potentially critical roles during embryo implantation [164]. These virus-induced alterations in the miRNA expression of endometrial cells might affect trophoblast cell behavior (Figure 2), supporting the hypothesis that HHV-6A might be associated with interference in correct implantation and pregnancy outcome [165].

The abilities of NK and endometrial cells have been described to be changed by HHV-6A infection. In fact, phenotypical and functional modifications of both endometrial NK (eNK) and epithelial cells have been reported in HHV-6A-positive infertile women samples, suggesting an imprint due to HHV-6A infection on both eNK cell immune-phenotype and receptors repertoire (Figure 2). In particular, during HHV-6A infection, eNK cells seem to acquire a cytotoxic profile as an attempt to limit the infection, which involves the NKG2D receptor [162]. The persistence of activated eNK and of subclinical HHV-6A infection could alter endometrial environment and disadvantage embryo implantation and placentation, and it could potentially have serious adverse side effects, such as pre-eclampsia, fetal growth restriction and stillbirth, as demonstrated by the increase in chemokines, mainly IP10 and FasL, in uterine flushing samples from HHV-6A-positive infertile women (Figure 2). 

In addition, Rizzo et al. observed a lower percentage of KIR2DL4-positive eNK cells in primary infertile women in correlation to the diminished expression of soluble HLA-G [166]. This evidence supports the potential role of HHV-6 in female diseases, as a consequence of HLA-G modulation, that can in turn induce anergy to eNK cells via the inhibitory KIR2DL4 receptor [166].

### 4.4. VZV

VZV is the etiological agent for chicken pox at time of primary infection, and it is usually associated to mild clinical course, but in pregnant women, it may occasionally lead to serious maternal and fetal diseases. Maternal VZV can infect the baby by different routes: (a) transplacental viremia, (b) ascending infection during birth or (c) respiratory droplet/direct contact with infectious lesions after birth.

Even if the primary mechanism of VZV transfer across the placenta remains unclear, it is postulated that infected T cells might be present in the decidua basalis [4], where both CD4+ and CD8+ T cells are reprogrammed by the virus, becoming more capable of crossing into the intervillous space [167].

However, reports vary on the histological features of VZV placental infection, suggesting that VZV could be transmitted to the fetus via the placenta without apparent viral replication within the placenta [168] (Figure 2).

Interestingly, nearly 20% of infants with intrauterine-acquired VZV primary infection develop neonatal or infantile zoster, usually with uncomplicated course [169]. The disease is thought to represent reactivation of the virus after primary infection in utero, and the short viral latency may be explained by the immature cell-mediated immune response in young children.

Moreover, recurrent chickenpox has been documented in pregnant women [170], underlining again the key role of the immune system. 

In addition to the tests of general antibody reactivity, tests of antibody avidity [171] and IgG isotype [172] can be used to assess the nature of VZV antibody responses. The avidity of antibodies seems to increase thereafter and during shingles, while there is a switch from IgM and IgG3 to IgG1 after primary disease [173] (Figure 2). Therefore, the clinical manifestation of these pregnant women can be due to the high virus load and low immune responses [174], whether the effect of pregnancy and associated hormones on VZV replication is not known.

VZV infection causes a very early release of IFN type I, which is particularly abundant at the lesion level [175] (Figure 2). NK cells can also be found early after VZV infection [176], suggesting their central role in controlling viral spread. In fact, while both cytotoxic NK and primed CD8+ T cells were nearly absent during the early phase of life-threatening primary VZV infection [177], their responses to VZV seem to be protective and associated to mild symptoms [178]. As an example, the detection of T cells within three days after the appearance of the varicella rash, with rapid host response to primary VZV infection, has been known to be associated with milder rash and a more rapid clearance of viremia in healthy subjects [176].

## 5. RNA Viruses and Gestational Infection

Viruses with an RNA genome pose a threat to global human health since they can cause severe pandemics and epidemics [179]. Many viral epidemics have occurred recently, putting vulnerable persons, including pregnant women, at risk. In fact, pregnant women who experience RNA virus infection, such as EBOLA virus, often went into worse results than the general population and non-pregnant women, reporting for example increased incidence of preterm labor and unfavorable fetal outcomes [180]. 

Maternal susceptibility to RNA viral infections is firstly due to an over- or under-activity of the maternal innate immune system. In a normal pregnancy, in contrast to the general inhibition of specialized immune cells, a global up-regulation of innate immune cells and effector mechanisms is observed [181,182]. Compared to the non-pregnant state, complement activity increases, and there is a substantial rise in circulating phagocytes and type I IFN-producing plasmacytoid DCs [17,183]. The innate pathways that drive anti-RNA-viral defense are specifically enhanced during pregnancy, according to longitudinal investigations of serial blood samples from pregnant women. For instance, IFN-induced STAT-1 activation, a crucial response to viral infection, rises in NK cells, monocytes and myeloid DCs throughout gestation [181,184].

**Figure 3:**
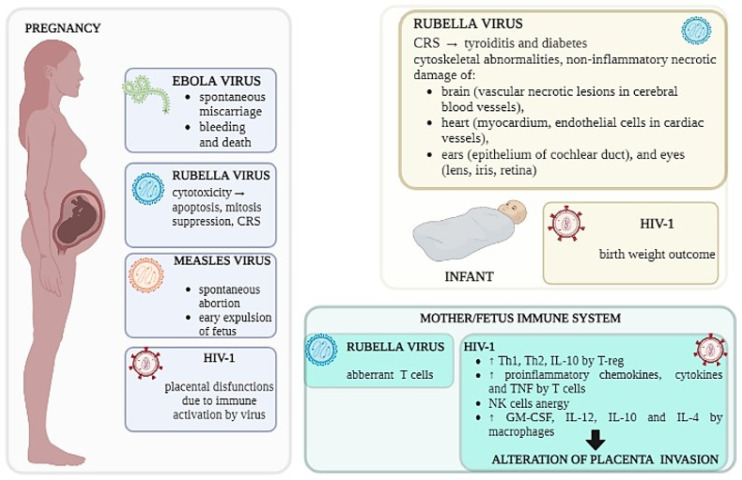
Representation of RNA viral infection and their effect in pregnancy, infants and mother/fetus immunity.

Among the most important pregnancy-related RNA virus infections, there are Rubella, HIV and emerging viruses such as Ebola, Zika, Dengue and SARS-CoV-2 infections, which are associated with an increased risk of spontaneous miscarriage, bleeding and death during pregnancy (Figure 3).

### 5.1. Rubella Virus

Dr. Norman Gregg described for the first time the catastrophic teratogenic consequences induced by rubella virus (RV), consisting of congenital cataracts and other malformations in the newborn following rubella (German measles) infection in the mother during pregnancy [185,186] (Figure 3). Despite the serious teratogenic effects reported, many RV cases are asymptomatic; thus, women of childbearing age are routinely tested for antibodies to RV in order to identify and immunize susceptible women. The vertical transmission of the infection depends on the gestational age at infection, leading to the development of congenital rubella syndrome (CRS), which is a condition that might affect any organ due to fetal non-lytic infections [185] (Figure 3), in which the virus can persist in different biological fluids for several months after infection [187]. 

First and early second trimesters of pregnancy seem to be the most susceptible times for CRS development. In fact, while there are few problems if the infection is transmitted to the fetus after 17 weeks of gestation, CRS affects nearly all fetuses infected in the first 8 weeks of pregnancy [185]. Deafness is the most prevalent of the several symptoms of CRS, while in the most critical cases, infected tissues from aborted fetuses showed extensive non-inflammatory necrotic damage to the brain (vascular necrotic lesions in cerebral blood vessels), heart (myocardium, endothelial cells in cardiac vessels), ears (epithelium of cochlear duct), and eyes (lens, iris, retina) [188] (Figure 3). Additional studies have shown that CRS is linked to autoimmune diseases and increases the risk of thyroiditis and diabetes [189] (Figure 3). 

Unfortunately, to date, little is known about the effect of RV on both the maternal and fetal immune system, and the few studies available reported an impaired cell-mediated immunity to rubella virus during pregnancy that could lead to congenital infection if it occurs early in utero, with a profound effect on the developing immune system [190,191,192]. 

It seems that rubella reinfection during pregnancy is not associated with a lack of neutralizing antibodies or persistent impairment of rubella-specific T responses. In particular, specific T response was present in almost cases of vertical transmission [191].

The specificity of a prenatal diagnosis, recommended when a maternal infection is diagnosed, is approximately 100% and is based on the detection of virus-specific-IgM in fetal blood or on the detection of the viral genome in amniotic fluid, fetal blood, or chorionic villus biopsies. The detection of RV-IgM by immunocapture ELISA, which has sensitivity and specificity that approach 100% in infected neonates, is the foundation for a postnatal diagnosis of congenital Infection. Regardless of whether a clinical manifestation of CRS is seen, it is crucial to perform a postnatal diagnosis of a congenital infection in order to give a specific follow-up treatment plan (including neurological and hearing monitoring) if an infection is found [187,193].

### 5.2. Measles Virus

Measles (rubeola) is a highly contagious respiratory disease brought on by a single-stranded, enveloped RNA virus that belongs to the Paramyxoviridae family, genus Morbillivirus [194].

Numerous studies have shown that pregnant women are more likely to suffer and die from measles than non-pregnant ones. In fact, even if measles virus (MV) in not teratogenic as rubella virus, it affects the physiological processes of immunotolerance that are present during pregnancy through alterations mainly on cell-mediated immunity that could result in spontaneous abortion or the early expulsion of the fetus [195] (Figure 3). Despite intensive vaccination campaigns that have been adopted in the most industrialized regions of the world, MV infection remains widespread, and the number of measles cases reported in the USA are rising, together with the chance of MV infection in pregnant women and the number of infections in women of reproductive age [196]. A study conducted on this outbreak showing the significant impact of the disease on pregnancy outcomes reported that 16% of pregnant women who underwent the infection had adverse outcomes probably correlated to increased CD8+ levels [197].

### 5.3. HIV

Despite highly active antiretroviral therapy (ART), HIV infection continues to be a concern for international health and for pregnancy as well. Currently, 81% of HIV-positive pregnant women worldwide are receiving ART and globally, the number of new perinatal infections has decreased by 50% because of increased ART use [198]. Even though the introduction of combination antiretroviral medication has significantly improved pregnancy outcomes and the health of children born to HIV-infected mothers, there are still worries about the effects of maternal HIV infection on pregnancy outcome and the health of HIV-exposed uninfected newborns [199].

One of the biggest changes from the immunological point of view that occurs during pregnancy and enables maternal–fetal tolerance is the increase in Th2 lymphocytes at the expense of Th1 [200,201], which is characterized by increased activated T cells and high levels of TNFs (Figure 3). Additionally, pregnant HIV-1-infected women’s placentas exhibit histological indicators of inflammation and an increased production of pro-inflammatory cytokines and chemokines [202] (Figure 3), which could impact the migration of T-cell subtypes to the placenta [203]. Additionally, a down-regulated expression of cytokine receptors, such as CD127 (IL-7 receptor) (Figure 3), highly expressed on tissue-resident T cells, could interfere with proper T-cell function.

NK cells are also necessary for placenta modeling vascularization [204]. This process is controlled by interactions between killer-cell immunoglobulin-like receptors (KIRs) expressed on maternal uterine NK cells and their corresponding HLA-C ligands on invading trophoblasts [205]. According to several studies, HIV-1 infection impairs NK cell function, resulting in the loss of effector functions and an increase in anergic NK cells, affecting the ability of uterine NK cells to support the invasion of maternal spiral arteries in the placenta [206,207] (Figure 3). Additionally, since HIV-1 infection has been linked to changes in chemokine receptors on immune cells, such as CCR5, the homing of NK cells to the placenta may be affected in HIV-1 infection [208]. Placental dendritic cells and macrophages in healthy pregnancy mostly display tolerogenic characteristics, as evidenced by a decreased expression of IL-12 and a high production of IL-10 and IL-4. Meanwhile, macrophages in HIV-1-infected women’s placentas have been seen to produce more GM-CSF and IL-2 while producing less IL-10 and IL-4 [209,210] (Figure 3). 

In addition to the RNA viruses mentioned so far, also, emerging RNA viruses have gained attention for their potential in causing complications during pregnancy and will be reviewed below.

## 6. Emerging Viruses and Pregnancy

### 6.1. Dengue Virus

Among the emerging viral infections representing a risk factor during the gestational period, Dengue Virus (DENV) infection is gaining more and more importance. Defining the risks associated with DENV infection in pregnancy has proved particularly challenging due to different reasons, such as the high proportion of asymptomatic cases and the difficulties associated with accurately diagnosing acute dengue fever (dengue IgM cross-reacts with other flaviviruses, and co-infections are common) [211]. The first to definitively establish the association between pregnancy and severe DENV infection have been Machado, comparing the outcomes in infected pregnant women to matched non-pregnant women of reproductive age, and they found an increased risk of severe dengue in the pregnant women (odds ratio 3.38) with a trend toward higher mortality (3 vs. 1.1%) [212]. Another study showed an 8.6-fold increase in the risk of postpartum hemorrhage in the presence of severe infection [213]. 

However, placental tropism, innate immune adaptations and the physiological increase in vascular permeability occurring in normal pregnancy could be at the base of this increased susceptibility to severe DENV infection in pregnancy [214].

**Figure 4:**
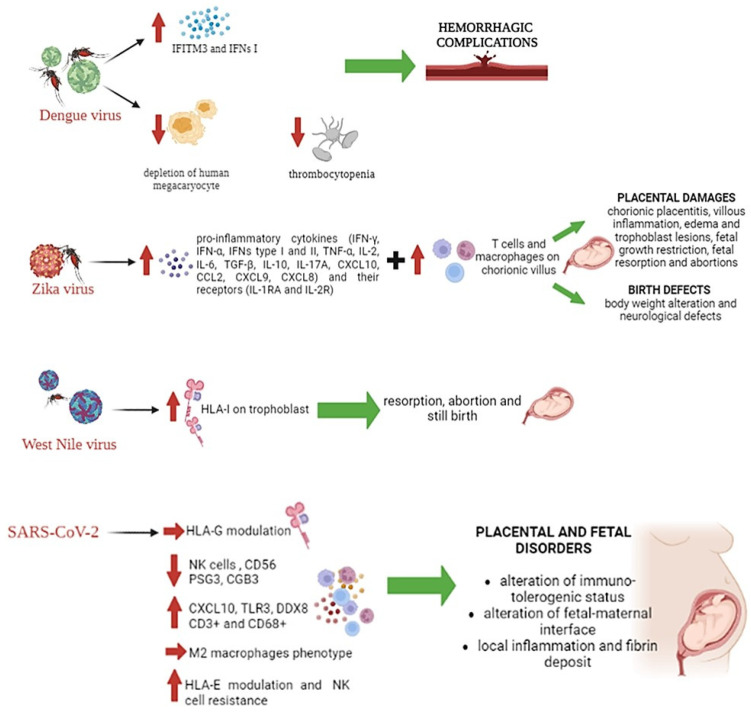
Representation of emerging viruses and their role in immune response alteration as cause of placenta and fetal disorders.

Some of the multiple effects of DENV infection include the depletion of human megakaryocytes that leads to dengue-induced thrombocytopenia (Figure 4). Campbell et al. found that DENV leads to a marked up-regulation of interferon-induced transmembrane protein 3 (IFITM3) on platelets, with a corresponding release of type I IFNs, and that the highest levels of IFITM3 expression correlated with the mildest disease [215,216] (Figure 4). It is conceivable that enhanced DENV-mediated megakaryocyte depletion, or a failure to up-regulate platelet IFITM3, could contribute to the higher rates of hemorrhagic complications during pregnancy.

However, type I IFN production is central to the innate anti-DENV response, although no specific studies have been conducted in pregnancy. In fact, DENV binds various PRRs, including RIG-I, endosomal TLR3 and endosomal TLR7, and the activation of these RNA sensors induces the deposition of Cb4 and C2a complement proteins on the virion surface, promoting the complement-mediated virolysis. Interestingly, emerging evidence states that vitamin D supplementation reduces cultured human DC susceptibility to DENV2 through the down-regulation of TLR3, TLR7 and TLR9 signaling [217], strengthening the importance of these sensors in the control of DENV infection. 

Again, it has been suggested that RNA interference is also involved in the activation of apoptosis in infected cells and is an important contributor to DENV defense as well [218].

### 6.2. Zika Virus

As already mentioned before, ZIKV infection during pregnancy has recently been associated with birth defects and abortions, mainly when infection occurs during the first and second trimesters of pregnancy [219,220,221,222] (Figure 4). 

Unfortunately, there is little information about the immune response to ZIKV infection in pregnant women [223]. During pregnancy, immunoglobulin synthesis is increased, whereas the cell-mediated response is decreased. These systemic changes are associated with an altered Th1/Th2 balance, with a prevailing anti-inflammatory Th2-like profile [224], that shifts toward a Th1/Th17 pro-inflammatory profile by the end of pregnancy [225], which could participate in viral pathogenesis (Figure 4).

The factors involved in ZIKV gestational pathogenesis are still unclear but may include prior exposure to closely related flaviviruses [226], a phenomenon originally described between DENV serotypes, which is known as antibody-dependent enhancement (ADE). 

It has been shown that the presence of DENV-specific antibodies in ZIKV-infected pregnant mice significantly increased placental damage, fetal growth restriction and fetal resorption [227] (Figure 4). This was associated with enhanced viral replication in the placenta, leading to an increased frequency of infected trophoblasts. 

ZIKV-infected human placental tissues also showed increased viral replication in the presence of DENV antibodies, which was reversed by FcγR blocking antibodies. Furthermore, ZIKV-mediated fetal pathogenesis was enhanced in mice in the presence of DENV-reactive monoclonal antibodies but not in the presence of the L234A and L235A (LALA) variant, indicating a dependence on FcγR engagement [227].

In a vertical-transmission model, ZIKV-immune plasma infused to timed pregnant mice increased fetal demise and decreased the body weight of the newborns [228] (Figure 4). Together, these data show that passive immunization with homotypic ZIKV antibodies, depending on the concentration, could either worsen or limit a subsequent ZIKV infection, suggesting the importance of immunity in the pathogenesis of this infection and underlining the key role exerted by antibodies.

Generally, pregnant women infected by ZIKV present chronic placentitis with chronic villous inflammation, edema, and trophoblastic lesions [229] (Figure 4). There is evidence that ZIKV infection compromises mesenchymal and Hofbauer cells in human villi [230], and further analysis reveals that ZIKV stimulates the proliferation of placental macrophages within the chorionic villous stroma [231] (Figure 4). CD163 or CD68-positive cells are co-localized with ZIKV antigens in vivo [30,232], as confirmed by ex vivo models showing a higher permittivity of placental macrophages to ZIKV than trophoblasts [233].

Although little is known about the cell-mediated immune response to ZIKV infection during pregnancy, a recent study reported a decreased frequency of granzyme B expressing total of CD8+ T cells in pregnant mice compared to non-pregnant mice [234], suggesting that the anti-ZIKV T-cell response quantity or quality may be reduced during pregnancy. It has been demonstrated that CD8+ T cells are necessary and sufficient to protect against systemic ZIKV challenge in both naïve and DENV-immune non-pregnant mice [235,236,237]. 

A similar requirement for CD8+ T cells against ZIKV was observed in the context of pregnancy prior to DENV exposure [235] together with a partially protective role for CD4+ T cells, suggesting that CD4+ T cell-mediated help may shape an optimal cross-reactive CD8+ T-cell response during the ZIKV infection of DENV-immune pregnant females. Alternatively, CD4+ T cells may exert their effect by regulating humoral immunity and the production of Th1 cytokines (IFN-γ, TNF-α, and IL-2) or CD4+ regulatory T cells could minimize pathology at the maternal–fetal interface [238].

In addition to this pro-inflammatory adaptive immune response, ZIKV infection also induces innate immunity activation as well. The innate immune response is mainly mediated by the induction of types I and III IFN, which induces an early autocrine antiviral stage in infected cells [239,240] (Figure 4). 

Again, also, high levels of IFN-γ, TNF-α, IL-6, TGF-β and IL-10 [241] have been observed during acute ZIKV infection in pregnant women [242,243] (Figure 4). The increases in IFN-α and IFN-γ suggest that pregnant women elicit effective antiviral responses [244,245], and the high levels of IL-10 suggest an antiviral response that could favor recovery and favorable evolution [246,247,248] (Figure 4).

### 6.3. West Nile Virus

Even though exposure to infected mosquitoes is the most important risk factor for West Nile Virus (WNV) infection, viral transmission includes blood transfusion [249], organ transplantation [250] and breastfeeding [251] as well as transplacental infection during pregnancy [252]. 

Transplacental WNV infection [253] is rare, since human placenta is a barrier which separates maternal and fetal circulations. However, as already mentioned for other viruses, it is not a perfect barrier, and in case of primary viral infections without a pre-formed maternal adaptive immunity, it can transmit the infection to the fetus.

Even if WNV was found in infants within a month of delivery from WNV-positive mothers, suggesting congenital transmission of the virus [253], little is known about the specific mechanisms exploited by WNV to cross the placenta, and it is unclear whether and how the virus is able to induce clinical manifestation in infants [253].

In vivo studies have reported an extremely high mortality rate in pregnant mice (98%, 60/61) compared with non-pregnant controls (52%, 28/53) independent of the infecting dose or the week of pregnancy [254]. Interestingly, although WNV RNA could be detected in both placentas and fetuses, antibody titers were similar between pregnant and non-pregnant mice and between surviving and non-surviving animals as well as WNV RNA titers in brains. In addition, from these observations in mice, it is possible to point out that pregnancy increases the risk of severe WNV infection and may help to understand the pathogenic mechanisms involved in WNV infection during human pregnancy [254]. 

Detailed studies of WNV infection of immunosuppressed human hosts, which are characterized by a similar immunotolerant condition as pregnancy, have highlighted important aspects of the disease pathogenesis during the gestational period as well [255]. While usually cleared within a week, severe infections in immunosuppressed patients have shown long-term chronic infection (months) of unknown pathogenesis [255,256].

As well as other viral infections, several innate immune escape strategies are exploited by WNV. These strategies a target key signaling IFNs response, which underscores the importance of bypassing this early immune response for effective viral replication [257]. 

Moreover, as well as being able to regulate HLA-I and/or II expression on many different types of embryonic cells [258,259,260], WNV infection is also able to induce HLA-I on primary trophoblast giant cells after 16 h of infection in the absence of type I or II IFNs [261] (Figure 4). This demonstrates that the block to HLA expression in the early embryo is not absolute and has effects on the recognition and eradication of viral disease by the maternal cellular immune system during early pregnancy. Thus, the embryo and placenta HLA expression early in gestation, together with concurrent viral antigen expression, could lead to the destruction of the embryo by both virus- and allo-specific maternal cytotoxic T cells. Indeed, there is a high incidence of resorption, abortion and stillbirth of embryos of several mammals due to transplacental infection by flaviviruses such as WNV [262], especially if the virus infects the woman during initial implantation [263] (Figure 4).

### 6.4. SARS-CoV-2

Several studies have investigated the possible multi-tissue tropism of SARS-CoV-2, basing on the wide expression of ACE2 and the proteases TMPRRS2, two of the most studied cellular receptors involved in viral entry by Spike interaction, in sites other than lungs [264]. 

A role for SARS-CoV-2 in pathological pregnancy has been firstly suggested by the finding of the co-expression of ACE2 and TMPRSS2 throughout gestation [265,266].

This suggested a possible effect on SARS-CoV-2 infection at both the placental and transplacental level. Moreover, other alternative receptors for SARS-CoV-2 entry into syncytiotrophoblast cells has been suggested, such as DPP4 (CD26) and CD147 [267]. A differential expression of both ACE2 and CD147 in the placenta of SARS-CoV-2-infected pregnant women compared to non-COVID19-infected pregnant women has been reported but without any direct evidence of viral transmission to the newborns [268,269].

Placental susceptibility to SARS-CoV-2 infection has been associated with a large debate concerning the occurrence of transplacental transmission. Although no transplacental transmission was initially reported, the first report of detection of SARS-CoV-2 in the placenta evidenced the presence of the virus only at syncytiotrophoblast levels and found local tissue inflammation and fibrin deposition [270] (Figure 4) in association with an inflammatory infiltrate of immune cells composed of CD3+ lymphocytes and CD68+ macrophages (Figure 4). The infiltration of CD68+ placenta macrophages in SARS-CoV-2-infected placenta was next confirmed [271,272], which was associated with an M2 phenotype in a case report of an asymptomatic COVID-19-positive woman [273] (Figure 4). Interestingly, Facchetti et al. reported a strong expression of SARS-CoV-2 Spike protein in areas with dense monocytes–macrophage inflammation, which suggested a local activation of these cells [272]. Thus, to date, although the presence of placental macrophages into placenta lesions was clearly established, their role in SARS-CoV-2 infection of the placenta remains to be elucidated.

However, a recent study reported a case of a 34-year-old woman admitted to the ICU due to severe COVID-19 infection whose newborn died by pulmonary embolism and thrombosis of the superior vena cava, in which immunohistochemical analysis demonstrated a SARS-CoV-2 NP presence in neonatal tissues [274]. 

As previously mentioned, placental infections might affect HLA-G expression, leading to placental and fetal disorders [155] (Figure 4), and the capability of SARS-CoV-2 to modulate HLA-G in its immune escape process has been confirmed [275,276,277,278]. 

It is established that during pregnancy, NK cells represent the main immune cell type in endometrial tissues (40% of the total leukocyte population, which increases to 60% and up to 75%) and show an increased degranulation response at the end of pregnancy, suggesting their role in inhibiting possible vertical transmission of infection [279]. However, although representing the first line of defense during viral infections is of extreme importance during pregnancy, NK cells have been reported to be anergic in COVID-19 patients [280]. A decrease in CD56-expressing immune cells, most associated with NK cells, has been observed [269] (Figure 4). The decrease in CD56 expression induces a cytotoxic phenotype that might alter the immune tolerogenic status at the fetal–maternal interface. Consequently, deficiencies and phenotypic alterations of CD56-positive NK cells might modify the placental homeostasis during SARS-CoV-2 infection with possible clinical implications on pregnancy [269] (Figure 4). Moreover, the mechanisms exploited by SARS-CoV-2 to modulate NK function have been evaluated [280], showing that the S1 protein is able to up-regulate HLA-E expression that induces cell resistance to NK cell lysis by interacting with the NK cell inhibitory receptor CD94/NKG2A (Figure 4) [281,282].

Interestingly, placenta samples delivered from SARS-CoV-2-positive subjects showed an increased expression of three immune genes: CXCL10, a pro-inflammatory chemokine secreted in response to interferon gamma, TLR3 and DDX58, involved in recognizing double-stranded RNA [268] (Figure 4), supporting the role of SARS-CoV-2 infection in modifying immune gene expression at the placenta level [283].

## 7. Vaccines and Antiviral Therapy in Pregnancy

A public health policy known as maternal immunization aims to protect pregnant women directly against the infectious disease in question as well as to give newborns and early children the best possible passive immunity. The best time to administer vaccinations is before conception, but there are some circumstances in which it is necessary to administer vaccines throughout pregnancy and postpartum [284].

Pregnant women who receive the vaccine develop vaccine-specific antibodies, which are passed to their unborn children through the placenta or through lactation. Only IgGs, among the various antibody isotypes, can cross the placenta starting from the 13th weeks of gestation, giving the fetus passive immunity. The fetal IgG levels then start to decline in the late second and early third trimester, showing a drop of up to 50% from the level reported in term children, supporting the timing of immunization in pregnancy to protect preterm infants [285]. The immune response peak resulting from a later vaccination (28–32 weeks) could closely match with the timing of maximal transplacental transfer of IgG and therefore potentially provide greater protection to the infant [285]. In fact, vaccination in the second trimester would provide longer exposure to maternal IgG, increasing the possibility of higher functional IgG in the infant [286]. To increase the proportion of pregnant women who receive vaccinations, future studies using various methodologies may offer a larger window of optimal vaccine scheduling. Pertussis, tetanus, diphtheria, polio, and the seasonal influenza vaccine are the only infections that can currently be vaccinated against while pregnant. However, future vaccines for pregnant women, including those for respiratory syncytial virus (RSV) and group B streptococcus (GBS), are now being developed [284]. There are live, inactivated, and multivalent monovalent and multivalent vaccines available for immunization. 

Live vaccines contain attenuated viruses that need to be multiplied for protective immunity to develop. As a result, this type of vaccine is contraindicated during pregnancy because the vaccine virus could theoretically spread to the fetus and endanger it [286]. To prevent this, women should wait one month before becoming pregnant after receiving a live vaccine.

Although it is not recommended during pregnancy, a live attenuated varicella vaccine was launched in 1995, and women who accidentally received it during pregnancy exhibited no issues [287]. However, because VZV vaccinations are contraindicated during pregnancy, it is advised that all women considering pregnancies be screened for varicella immunity and that non-immune women be immunized before becoming pregnant. On the other hand, postpartum vaccinations for non-immune pregnant women are advised [287].

The dengue infection has a similar live attenuated tetravalent vaccination. The live-attenuated flavivirus vaccine (CYD-TDV), which was approved in December 2015 for use in people as young as 9 years old [288,289], expresses the structural antigens of DENV. These antigens serve as the targets of the host immune response by inducing innate immune cells, neutralizing antibodies, and T-cell responses. Considering the potential danger of virus transmission to the fetus, CYD-TDV is contraindicated in pregnant women, just like the other live attenuated vaccines. Preclinical in vivo animal investigations have not identified any teratogenic effects in the offspring [290], but no assessments have been carried out in people. Further research is therefore required to determine the safety of dengue vaccine during pregnancy.

In contrast to live attenuated vaccines, mRNA–lipid nanoparticle (LNP) COVID-19 vaccines currently lack safety information for use during pregnancy, albeit in rare circumstances, severely vulnerable pregnant women have been evaluated. However, there are many justifications for vaccination during pregnancy that support the safety of COVID-19 vaccinations. The primary factor contributing to these vaccines’ promising safety is that they are made of mRNA encapsulated in a lipid nanoparticle, which prompts the host to produce coronavirus spike proteins, thereby inducing the production of antibodies [291] without containing live viruses or adjuvants that could harm a fetus in development [292]. 

Additionally, the HPV vaccine is a vaccination that is gaining popularity during pregnancy. The virus-like particles (VLPs) in HPV vaccines are recombinant and augmented by an adjuvant, which stimulates a stronger immune response than a natural infection [293]. 

Even though giving the vaccine to pregnant women is not advised [294], it commonly happens accidentally because most recipients are fertile young women. The recommended protocol is to postpone the three-dose regimen until after the fetus is fully developed if a woman is discovered to be pregnant while receiving an HPV series. Government regulations, however, do not apply to pregnancy testing before immunization because they could impede compliance and have a detrimental impact on vaccine uptake by delaying vaccination. 

The chances of spontaneous miscarriage, serious birth defects, stillbirth, and preterm birth were not shown to be significantly greater with quadrivalent HPV vaccination during pregnancy in a large, statewide study conducted in Denmark [295]. Although no unfavorable outcomes of the pregnancy have been linked to vaccination, it is nevertheless important to properly demonstrate the safety of vaccination in pregnant women and, in the meantime, avoid its administration. 

The emergency vaccination against hepatitis A and B during high-risk pregnancy has been accepted [296] in addition to the administration of influenza, tetanus, diphtheria, and pertussis vaccines [297], since the administration of other vaccines to pregnant women should be based on whether the benefits outweigh the risks [298]. Recombinant HB vaccines are the only type of HBV vaccination that is widely available, safe for newborns [299,300] and adults [301,302] and efficacious compared to plasma-derived vaccines. According to studies [303,304,305,306,307], HBV vaccination during pregnancy is immunogenic and causes a passive transfer of antibodies to the fetus. 

However, one study looked at the timing of vaccination and found that babies born to moms who had their vaccinations during pregnancy had lower anti-HBs levels than babies born to mothers who received their vaccinations prior to becoming pregnant [308]. Therefore, based on this observation, it may be advised that women who are planning a pregnancy receive their shots before conception. This will improve the benefits for the unborn child and reduce the possibility of unfavorable pregnancy outcomes caused by vaccine safety.

The clinical trials of various medicines and antiviral medications, however, rarely include pregnant women in their cohorts due to ethical considerations, as has been noted for vaccine safety studies [308]. Therefore, even though the danger in animals is not always a reliable indicator of the same risk in people [309], animal studies are occasionally the only source to evaluate how experimental medical interventions would harm the fetus [310]. Because there are not any clinical trials conducted while pregnant, the safety of a medicine for pregnant women can only be determined after it is sold and used.

Acyclovir and zidovudine are the two first antiviral medications to be introduced because of the significant morbidity of the genital herpes simplex virus and HIV epidemics [311]. Since then, their effectiveness and safety have been established in non-pregnant patients, but use in pregnancy has been prohibited due to several fetus-related issues. Acyclovir and zidovudine have only been used during pregnancy in case reports and pharmacokinetic investigations [312].

Acyclovir reduces viral DNA replication by selectively inhibiting human HSV DNA polymerase, whereas zidovudine inhibits HIV-1 reverse transcriptase, which is an enzyme involved in a crucial stage of the HIV life cycle. ZDV’s antiviral effect depends on the drug’s intracellular conversion to a triphosphate metabolite, which needs host cell kinases to occur [313]. The data are currently sparse, and there are no prospective controlled studies, even though the body of research on the safety of oral acyclovir and valacyclovir does not show that these two antiviral medications have a deleterious impact on the fetus [313]. 

However, because of the encouraging data that have already been presented, doctors are permitted to treat pregnant patients with acyclovir or valacyclovir for primary or recurrent HSV infection, treating the mother while also reducing viral transmission without harming the fetus [313]. Moreover, as was the case with HSV and HIV, managing chronic hepatitis B (CHB) during pregnancy while using antiviral medications is a difficulty that could have negative effects on both the mother and fetus. The impact of antiviral medication during pregnancy for maternal liver illness and the prevention of perinatal transmission are important factors to consider and research. However, antiviral therapy can only very rarely be used during pregnancy [314]. Most pregnant women in the immune-tolerance period should be observed without treatment [311,315]. Antiviral medication may be started during pregnancy or continued if it was started before pregnancy, for example, if pregnant women have an acute hepatitis flare-up or have underlying severe or advanced liver disease, such as cirrhosis [316].

In contrast to the antiviral therapies previously mentioned, the novel perspective suggested for the treatment of hepatitis is that the potential risks of fetal exposure to antiviral medications should not prevent the start of therapy, as it may be lifesaving for both mother and fetus. The preferred medication appears to be tenofovir (TDF), which functions as a nucleotide reverse transcriptase inhibitor, and it should be continued throughout the pregnancy [317]. 

Additionally, among the various approaches that have been suggested to treat COVID-19 patients, specific antiviral therapies [318] such as nucleoside analogs, which inhibit reverse transcription and are the most effective antiviral agents currently on the market against SARS-CoV-2 infection [319,320], play a crucial role. Remdesivir efficacy and safety, for instance, have been assessed in pregnant women with coronavirus disease 2019 (COVID-19), with the clinical condition improving mostly in those who had a better clinical status at baseline and received remdesivir treatment earlier. Even though most deliveries by cesarean section were urgent cases and no vertical transmissions were reported, transaminitis was a side effect that was noticed [321,322,323,324,325]. Remdesivir’s effectiveness and safety profile in treating pregnant women with COVID-19 are therefore still unknown, and more research is required.

The current therapy choices for flavivirus infections are largely supportive care-based (antipyretics, analgesics, and antihistamines). ZIKV and dengue are similar enough that some antiviral treatments that work for both infections can be investigated [326]. Clinical trials in dengue-infected humans have already evaluated a few drugs, including chloroquine, balapiravir, and celgosivir, whose mechanisms of action include immunomodulatory activity, nucleoside inhibition, and host-glucosidase inhibitor, respectively. Unfortunately, there was no proof that the plasma viremia had been effectively reduced [327], and the drug’s possible teratogenicity or toxicity should be carefully considered [326].

## 8. Conclusions

Immunological modifications during pregnancy allow maternal tolerance of the semi-allogeneic fetus while simultaneously increasing maternal vulnerability to infection. The processes that contribute to maternal and fetal harm caused by viral infections are complicated and heavily reliant on pathogenesis variables such as viral susceptibility, tissue and cellular tropism, and host–pathogen interactions in the placenta niche. The placenta is a physical and immunological barrier that protects the fetus from infections in the maternal blood. Nevertheless, certain pathogens, particularly viruses, can enter the fetal compartment via hematogeneous or decidual spread, causing congenital disorder and eventually fetus death. Previous epidemics of numerous new viral infections have resulted in poor pregnancy outcomes, including maternal and fetal morbidity and death, as well as peripartum infections with severe sequelae, even in the absence of maternal-to-fetal transmission. The major change around the subset of immunity cells at the maternal–fetal interface regard natural killer (NK) cells, macrophages, dendritic cells (DCs) and T cells, whose function and secretome can be changed during viral infections, potentially leading to preterm birth, congenital malformations, abortion, or stillbirth. These findings highlight the necessity of understanding the mechanisms through which viruses can reach the placenta or can lead to negative outcomes. Considering the success of tetanus vaccination during pregnancy, recommendations for immunization during gestation against several infectious diseases have increased. Given the advances in targeted drug delivery using nanoparticles (NPs), several research groups are currently focusing on developing virus-like particles (VLP) or NPs meant to be used for gestational active immunization [284]. Since the research and development of VLPs and NP presents an opportunity for effective pregnancy therapy, their translation into healthcare settings must go through a lengthy and challenging toxicity testing process. Virus-like particles (VLPs) or NPs are supramolecular entities that resemble viruses but do not have the potential to infect. VLP/NP may be customized to target specific immune system cells while simultaneously presenting antigens to the immune system, making them attractive vaccination candidates [285]. For example, wild-type SARS-CoV-2 and group-specific antigen (Gag) VLPs with a diameter of around 145 nm represent very interesting alternatives for the development of novel coronavirus vaccine candidates [286]. Similarly, Garg and colleagues [287] recently published a new VLP-based Zika virus vaccination that was proven to induce neutralizing antibodies postimmunization. The preliminary experiments, however, were performed in nonpregnant mice, and no data on the VLP influence on pregnancy were presented. Differences in the real physiology, anatomy, and uterine environment, as well as the cost of conducting studies on mouse models, limit the utility of such models. Therefore, new promising strategies for studying VLP have been developed, which are based on reproducing the mother–fetus interface on a chip, organ-on-chip (OoC) [288].

Despite all the evidence, essential research on the development of safe antiviral medicines and vaccinations that prompt maternal immune response should be promoted.

## Figures and Tables

**Table 1:** Summary of the possible outcome of infection during pregnancy, for both mother and fetus.

	Infection of Placenta without Infection of the Fetus	Fetal Infection without Placental Infection	Absence of Fetal and Placental Infection	Infection of Placenta and Fetus
Microorganism interaction	Microorganisms reach the intervillous space of the maternal side on placenta. They do not spread to the fetus.	Microorganisms can cross directly through the chorionic villi, using pinocytosis or diaphysis mechanisms.	Interaction of microorganisms that might reach the fetus with placenta.	Microorganisms pass from the infected placenta to the fetal compartment through the chorionic villi, directly infecting fetal membranes and amniotic fluid.
Effects	The infection does not involve the fetus thanks to fetal defense mechanisms by placental macrophages and the local production of antibodies and cytokines.	Infection of maternal leukocytes and erythrocytes.	The fetus is protected from the attack of microbial agents by the maternal endothelial reticular system and circulating leukocytes.	Transplacental transmission of infection can lead to death, with a subsequent resorption of the embryo, intrauterine fetal death or premature term birth of infected infant.

## Data Availability

All the data are reported in the manuscript.
